# Finding *trans*-regulatory genes and protein complexes modulating meiotic recombination hotspots of human, mouse and yeast

**DOI:** 10.1186/s12918-014-0107-1

**Published:** 2014-09-11

**Authors:** Min Wu, Chee-Keong Kwoh, Xiaoli Li, Jie Zheng

**Affiliations:** 1 Institute For Infocomm Research, A*Star, 1 Fusionopolis Way, Singapore 138632, Singapore; 2 School Of Computer Engineering, Nanyang Technological University, Singapore 639798, Singapore; 3 Genome Institute Of Singapore, A*Star, Biopolis, Singapore 138672, Singapore

**Keywords:** Meiotic recombination hotspots, Trans-regulators, Protein-protein interactions (PPI), Random walk, Protein complexes, Gene Ontology (GO) term analysis, Epigenetic functions

## Abstract

**Background:**

The regulatory mechanism of recombination is one of the most fundamental problems in genomics, with wide applications in genome wide association studies (GWAS), birth-defect diseases, molecular evolution, cancer research, etc. Recombination events cluster into short genomic regions called “recombination hotspots”. Recently, a zinc finger protein PRDM9 was reported to regulate recombination hotspots in human and mouse genomes. In addition, a 13-mer motif contained in the binding sites of PRDM9 is found to be enriched in human hotspots. However, this 13-mer motif only covers a fraction of hotspots, indicating that PRDM9 is not the only regulator of recombination hotspots. Therefore, the challenge of discovering other regulators of recombination hotspots becomes significant. Furthermore, recombination is a complex process. Hence, multiple proteins acting as machinery, rather than individual proteins, are more likely to carry out this process in a precise and stable manner. Therefore, the extension of the prediction of individual *trans*-regulators to protein complexes is also highly desired.

**Results:**

In this paper, we introduce a pipeline to identify genes and protein complexes associated with recombination hotspots. First, we prioritize proteins associated with hotspots based on their preference of binding to hotspots and coldspots. Second, using the above identified genes as seeds, we apply the Random Walk with Restart algorithm (RWR) to propagate their influences to other proteins in protein-protein interaction (PPI) networks. Hence, many proteins without DNA-binding information will also be assigned a score to implicate their roles in recombination hotspots. Third, we construct sub-PPI networks induced by top genes ranked by RWR for various species (*e.g.*, yeast, human and mouse) and detect protein complexes in those sub-PPI networks.

**Conclusions:**

The GO term analysis show that our prioritizing methods and the RWR algorithm are capable of identifying novel genes associated with recombination hotspots. The *trans*-regulators predicted by our pipeline are enriched with epigenetic functions (e.g., histone modifications), demonstrating the epigenetic regulatory mechanisms of recombination hotspots. The identified protein complexes also provide us with candidates to further investigate the molecular machineries for recombination hotspots. Moreover, the experimental data and results are available on our web site http://www.ntu.edu.sg/home/zhengjie/data/RecombinationHotspot/NetPipe/.

## 1 Background

Recombination is one of the most fundamental processes in molecular biology [1]. It is a process that homologous chromosomes exchange their arms and such crossover events tend to occur more frequently within some short regions called “recombination hotspots”. The understanding of the mechanisms for recombination hotspots would thus shed light on various important aspects in molecular biology and medicine, such as genome instability, birth-defect diseases, disease gene mapping, molecular evolution and so on [2].

Recently, there has been much progress in the discovery of the mechanisms for meiotic recombination hotspots in mammalian genomes. For example, a zinc finger protein PRDM9 was reported as a *trans*-regulator of recombination hotspots in human and mouse genomes [3]-[5]. PRDM9 binds to DNA and its binding site contains a 13-mer motif previously found to be enriched in human hotspots [6]. In [7], Smagulova *et al.* analyzed the molecular features of mouse recombination hotspots and observed that a consensus motif enriched in mouse hotspots aligns with the predicted binding site of mouse PRDM9 significantly. Using an LD-based approach named LDsplit, Zheng *et al.*[2] identified HapMap SNPs (single nucleotide polymorphisms) as *cis*-regulators of recombination hotspots. In addition, the authors [2] also found an enriched 11-mer motif which closely matches the aforementioned 13-mer motif bound by PRDM9 and enriched in human recombination hotspots.

Although significant breakthroughs have been made in the understanding of the regulatory mechanisms of meiotic recombination hotspots, they are mainly focused on the well-known protein PRDM9. However, it is estimated that PRDM9 can explain only 18% of variations in human recombination phenotype [3]. Meanwhile, the 13-mer motif contained in the binding sites of PRDM9 covers only 41% of human recombination hotspots [6]. Therefore, PRDM9 is unlikely to be the only *trans*-regulator of recombination hotspots and we are highly motivated to discover other genes as *trans*-regulators. Recombination is such a complex process that it is unlikely to be regulated by individual proteins. Rather, multiple proteins need to act in concert as a molecular machinery to carry out the process precisely and stably, *e.g.*, the Mre11 complex with Mre11, Rad50 and Nbs1 (also known as MRN complex) in yeast [8] and a FIGNL1-containing protein complex with FIGNL1 and SPIDR in human [9]. As such, the extension of the prediction from individual proteins to protein complexes is highly desired. Furthermore, the function of PRDM9 for regulating recombination hotspots is well conserved among human, chimpanzee and mouse [3],[4]. It would be an interesting question in comparative genomics whether there are any other genes or complexes whose functional roles in regulating recombination hotspots are conserved among species.

**Figure 1:**
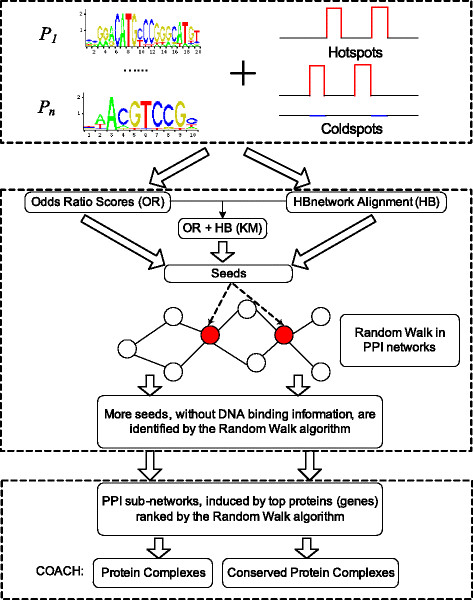
**The flowchart of our pipeline to identify genes and protein complexes associated with recombination hotspots.** Figure 1 shows the flowchart of our pipeline to identify genes and protein complexes associated with recombination hotspots. In particular, the inputs are the binding motifs of DNA-binding proteins, hotspots, as well as coldspots generated by ourselves. Individual genes will be identified (1) by various prioritizing methods, *i.e.*, OR, HB and KM, using the binding information between genes and hotspots, (2) by the Random Walk with Restart (RWR) algorithm on the PPI networks. Protein complexes and conserved protein complexes are detected from the PPI subgraphs induced by the individual genes that are ranked top by the RWR algorithm.

To address the above issues, this paper introduces a pipeline as shown in Figure 1 to identify genes and protein complexes associated with recombination hotspots. First, we introduce two complementary methods, *i.e.*, Odds Ratio scores (OR for short) [10] and Hotspot-Binding (HB) network alignment method [11], to prioritize genes as candidates of *trans*-regulators. In addition, we propose a novel method (called KM in Figure 1) to combine the results from both OR and HB methods. Second, using genes identified by the above prioritizing methods as seeds, we apply the Random Walk with Restart algorithm (RWR) to propagate the influences of these seeds to other proteins in protein-protein interaction (PPI) networks. As such, many proteins without DNA-binding information will be assigned scores to implicate their roles in recombination hotspots. Third, we construct sub-PPI networks induced by top genes ranked by RWR for various species (*e.g.*, yeast, human and mouse). We further detect conserved protein complexes from those PPI sub-networks, which may perform functions related to recombination hotspots.

In order to evaluate the results of our pipeline, we utilize multiple perspectives of GO term analysis. First, the GO term enrichment analysis shows that epigenetic functions are enriched in the seeds selected by various prioritizing methods. Second, we calculate the semantic similarity between identified genes and existing recombination related GO terms (*i.e.*, GO:0006310—DNA recombination and GO:0007126—Meiosis). Genes top-ranked by RWR are demonstrated to have even higher similarities to these two particular terms than the seeds in human and yeast. This shows RWR in PPI networks is a credible complement to the existing methods since it enables the detection of novel genes without binding information. Lastly, in contrast to most existing methods which only explore the individual genes, in this paper we carry out analysis at protein-complex level and capture the underlying modularity and functional organization among those recombination related proteins.

## 2 Methods

### 2.1 Prioritizing genes for recombination hotspots

We first briefly introduce two different methods proposed in our previous studies for prioritizing genes for recombination hotspots, namely the Odds Ratio method (OR) [10] and HB network alignment method (HB) [11]. Moreover, we present a graph-matching based method (the KM method as shown in Figure 1) in this paper to combine the results of the above two methods. As such, we can identify *trans*-regulators with these methods from different perspectives and later on we will conduct comprehensive comparisons among them. In addition, the terms “gene” and “protein” are used interchangeably in this paper.

#### 2.1.1 Odds Ratio scores

Given a transcription factor (TF) with a binding motif, we are able to count the occurrences of this motif in hotspots and coldspots using the FIMO software [12]. We measure the preference of the TF to bind in hotspots with the Odds Ratio *O*_*hc*_=(*HM*/*HN*)/(*CM*/*CN*). Here, *HM* is the number of hotspots with at least one motif occurrence (*i.e.* a hit of FIMO search), *HN* is number of hotspots without any hit (*i.e.HN*=*N*_*H*_−*HM*, where *N*_*H*_ is the number of hotspots), *CM* is the number of coldspots with at least one hit, and *CN* is the number of coldspots without any hit (*i.e.CN*=*N*_*C*_−*CM*, where *N*_*C*_ is the number of coldspots). We predict those TFs with high Odds Ratio scores, *i.e.*, those preferring to bind to hotspots rather than coldspots, as candidates of *trans*-regulators of recombination hotspots. For more information, please refer to our previous study [10] on the Odds Ratio scores for TFs.

#### 2.1.2 HB network construction and alignment for TFs

We collect the Hotspot-Binding profiles (HB profiles) for TFs. In particular, we divide the whole genome into *λ* bins with fixed length (*e.g.*, 5M bases) and the HB profile of a TF *g* is represented as a *λ*−dimension vector, *HB*(*g*)=(*b*_1_,*b*_2_,⋯,*b*_*λ*_), where *b*_*i*_ is the number of hotspots in the *i*^*th*^ bin that *g* binds to. Subsequently, we can build a HB network for TFs, where a node is a TF and an edge between two TFs indicates they have similar HB profiles. The similarity between two HB profiles is measured by Pearson correlation coefficient. Two TFs are connected in the HB network when the similarity between their HB profiles is larger than a pre-defined threshold (*e.g.*, 0.7 is used in this paper).

We construct HB networks for multiple species and apply a network comparison toolkit named NCT [13] to align these networks. NCT will output the conserved subgraphs among HB networks. Such cross-species alignment of HB networks can detect evolutionarily conserved network motifs associated with recombination hotspots, which are believed to be more significant than signals from single-species analysis [13],[14]. Furthermore, it is observed that proteins involved in multiple modules tend to be more biologically important [15]. Therefore, for those TFs in HB networks, we evaluate their relevance to recombination hotspots based on their frequency in the conserved clusters collected by NCT. More specifically, for a TF *g*, its relevance score *R*(*g*) to recombination hotspots is measured by its frequency in the conserved clusters, *i.e.*, the number of conserved clusters involving *g*, normalized by the maximum frequency over all the genes. Similar to the Odds Ratio scores, we use the above relevance scores to rank candidate genes related to recombination hotspots.

#### 2.1.3 KM method to combine results from OR and HB

Given *n* TFs and two rankings *σ*_*u*_ and *σ*_*v*_ for these TFs, the Spearman’s Footrule distance, $F\left(\sigma_{u},\sigma_{v}\right)=\overset{n}{\underset{i=1}{\sum }}|\sigma_{u}(i)-\sigma_{v}(i)|$, reflects the consistency between these two rankings [16]. Here *σ*_*u*_(*i*) is the position of the *i*^*th*^ TF in the ranking *σ*_*u*_. For example, *σ*_*u*_(*i*)=1 means that the *i*^*th*^ TF is in the top position of *σ*_*u*_. Assume that *σ*_*o*_ and *σ*_*h*_ are two rankings for TFs from OR and HB, respectively. Our objective is to find a new ranking *σ*^∗^ in Equation 1 which has minimal distance to both *σ*_*o*_ and *σ*_*h*_, *i.e.*, maximal consistency with both *σ*_*o*_ and *σ*_*h*_.

(1) $$\sigma^{*}=\arg\underset{\sigma }{\min}\overset{n}{\underset{i=1}{\sum }}|\sigma_{o}(i)-\sigma (i)|+|\sigma_{h}(i)-\sigma (i)|.$$

We build a weighted complete bipartite graph *TG*=(*T*,*P*,*w*), where *T* containing nodes on one side is the set of TFs and *P* containing nodes on the other side denotes the positions from 1 to *n*. There is an edge between *t*∈*T* and *p*∈*P*, denoting a possible assignment of *t* to rank *p*. The weight *w*(*t*,*p*)=|*σ*_*o*_(*t*)−*p*|+|*σ*_*h*_(*t*)−*p*|, denotes the footrule distance between existing rankings (*σ*_*o*_ and *σ*_*h*_) and a possible ranking that places the TF *t* at the position *p*. As such, we solve the problem in Equation 1 by finding a minimum weighted matching in *TG* using Kuhn-Muntres algorithm [16],[17], and we denote this combining strategy as KM method for short.

### 2.2 Random walk in PPI networks

*Trans*-regulators can be predicted by the above prioritizing methods, *i.e.*, OR, HB and KM. However, the power of these methods would be limited due to the small number of TFs with known binding motifs. For example, out of tens of thousands of known human and mouse genes, there are only 158 binding motifs for human and 148 for mouse in two well-known databases (*i.e.*, JASPAR [18] and TRANSFAC [19]), respectively. Meanwhile, a large amount of protein-protein interaction (PPI) data are available and they are often modeled as graphs, where nodes are proteins and edges are interactions between proteins, for predicting novel protein interactions [20], protein functions [21], protein complexes [22], disease genes [23],[24] etc. In this work, we exploit PPI data to evaluate the relevance of genes (proteins) to recombination hotspots, by a Random Walk with Restart algorithm (RWR) [23],[25].

RWR simulates a random walker, which starts on a set of seed nodes and moves to their neighbors randomly at each step. Therefore, RWR propagates the influence from the seed nodes to the remaining nodes in the PPI network and can be used to measure the proximity of other nodes to the seed nodes. Let *p*_0_ be the initial vector showing the relevance of seeds to recombination hotspots (*i.e.*, assigned by our prioritizing methods) and *p*_*t*_ be a vector in which the *i*-th element shows the relevance of node *i* at step *t*. The relevance vector at step *t* + 1 is then calculated as

(2) $$p_{t+1}=(1-\gamma )\times W\times p_{t}+\gamma \times p_{0},$$

where *W* is the transition matrix of the PPI network and each element *W*_*ij*_ is the transition probability from node *i* to node *j*. In this paper, the normalized adjacency matrix of the PPI network is considered as the transition matrix. The parameter *γ*∈(0,1) is the restart probability. At each step, the random walker may return to seed nodes with probability *γ*. In our experiments, it is set as 0.7 (the same as the setting in [23]). *p*_*∞*_(*i*) is the final relevance of node *i* to recombination hotspots. We can obtain the relevance vector at the steady state (*p*_*∞*_) efficiently by performing iterations until the difference between *p*_*t*+1_ and *p*_*t*_ is below a threshold, for example, 10^−10^[23].

Based on the RWR algorithm in PPI networks, genes that are highly interactive with the seed genes will accumulate more influence pumped from the seeds. Hence, we can consider them as novel genes associated with recombination hotspots even if they may not have known DNA binding motifs.

### 2.3 Predicting protein complexes for recombination hotspots

After prioritizing genes associated with recombination hot-spots, we construct sub-networks for various species, which are induced by those top-ranked genes (*e.g.*, top 200 genes [26]). Therefore, we detect protein complexes highly related to recombination hotspots from these PPI sub-networks using the COACH algorithm [27], which predicts dense regions in PPI networks as protein complexes. Furthermore, we can detect evolutionarily conserved protein complexes involved in recombination hotspots as follows.

Let *H*={*H*_1_,⋯,*H*_*m*_} and *M*={*M*_1_,⋯,*M*_*n*_} be the sets of protein complexes predicted by COACH from sub-networks of PPI networks in two different species (*e.g.*, human and mouse) respectively. Then, we build a bipartite graph *CG*=(*H*,*M*,*E*,*w*), where *H* and *M* represent two sets of super-nodes (*i.e.*, proteins are nodes and predicted protein complexes thus are considered as super-nodes in the bipartite graph *CG*) and the edge weights are defined using the neighborhood affinity (NA) score [27],[28] in Equation 3. Here, |*H*_*i*_∩*M*_*j*_| is the number of ortholog pairs between *H*_*i*_ and *M*_*j*_.

(3) $$w(H_{i},M_{j})=\frac{|H_{i}\cap M_{j}{|}^{2}}{\left|H_{i}|\times |M_{j}\right|}.$$

In previous studies [27],[28], two protein complexes with many common proteins, which have a NA score larger than or equal to a threshold (generally set as 0.25), will be considered as the same protein complex. Similarly, a pair of super-nodes (*i.e.*, protein complexes) in our bipartite graph *CG* with an edge weight larger than or equal to the threshold will be considered as a pair of conserved complexes and all the edges with weights lower than the threshold will be removed from *CG*. Obviously, the weight here indicates the conservation between the complexes from two species. To maximize the conservation between two species [29], we detect conserved protein complexes by finding maximal weighted matching in *CG* using Kuhn-Muntres algorithm [17]. Finally, our conserved protein complexes are those pairs in the obtained maximum weighted matching.

### 2.4 GO term analysis

Given a gene *g*, *T*(*g*) is the set of GO terms annotating this gene. We define the similarity between a term *t* and a gene *g*, *S*(*t*,*g*), in equation 4 and subsequently define the similarity between *t* and a set of genes *V*, *S*(*t*,*V*), in equation 5.

(4) $$\begin{array}{lcrr} S(t,g) & = & \underset{t^{\prime }\in T(g)}{\max}\text{sim}\;\left(t,t^{\prime }\right) & \end{array}$$

(5) $$\begin{array}{lcrr} S(t,V) & = & \frac{1}{\left|G\right|}\underset{g\in V}{\sum }S(t,g) & \end{array}$$

Here, *sim* (*t*,*t*^′^) in equation 4 is the semantic similarity between GO terms *t* and *t*^′^ and we applied the method in [30] to calculate *sim* (*t*,*t*^′^).

Let *HG* denote the set of genes selected by our prioritizing methods while *G* is the whole set of TFs with binding motifs. Now, *S*(*t*,*G*) and *S*(*t*,*HG*) can be utilized to show the term *t*’s enrichment in the gene group *G* and *HG*, respectively. Therefore, the gap score for the term *t*, *gap*(*t*) in equation 6, can be used to discriminate *t*’s enrichment in *HG* and *G*. For example, a large gap indicates that *t* is enriched in the *HG* genes while not enriched in the whole gene group *G*.

(6) $$\text{gap}(t)=\frac{S(t,\text{HG})-S(t,G)}{S(t,G)}$$

## 3 Results and discussions

### 3.1 Experimental data

In this section, we briefly introduce the data used in our experiments. 3,600 yeast recombination hotspots were collected from [31]. 39,551 human recombination hotspots were estimated from HapMap genetic map by the LDhat package [32]. In addition, 9,874 mouse recombination hotspots were downloaded from [7]. DNA sequences for yeast (version: sacCer3), mouse (version: MGSCv37) and human (version: GRCh37) were downloaded from NCBI.

To calculate the Odds Ratio scores and collect the HB profiles, the binding motifs of TFs were downloaded from JASPAR and TRANSFAC databases. After processing, we obtained 177 binding motifs of yeast, 158 of human and 148 of mouse, respectively. Yeast PPI data, with 17,201 interactions among 4,930 proteins, were downloaded from the DIP database [33]. Human PPI data were downloaded from the BioGRID database [34], consisting of 11,120 proteins and 55,014 interactions among these proteins. Mouse PPI data were downloaded from [35], with 10,348 proteins and 63,882 interactions. Lastly, the GO data for various GO term analysis were downloaded from http://www.geneontology.org.

### 3.2 Genes ranked by various prioritizing methods

#### 3.2.1 Genes ranked by Odds Ratio scores

**Figure 2:**
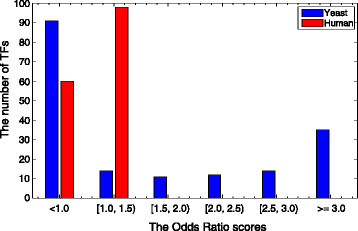
**The distributions of the Odds Ratio scores for TFs in human and yeast.** Figure 2 shows the distributions of the Odds Ratio scores for TFs in human and yeast, respectively. For example, 35 yeast TFs have scores larger than 3.0 and 14 have scores in the range (2.5, 3.0], while 98 human TFs have OR scores in the range [1, 1.5) and 60 with OR scores lower than 1. Note that all the human TFs have OR scores lower than 1.5.

Next, we show the properties of the genes with high Odds Ratio scores in yeast and human by GO term analysis (the results for mouse have already been shown in [36]). In Figure 2, we observe that the distributions of the Odds Ratio scores for TFs in yeast and human are quite different. For example, there are 35 TFs in yeast with scores larger than 3.0 and 14 with scores in the range (2.5, 3.0], while all the human TFs have scores less than 1.5. Therefore, we cannot select the set of genes with high Odds Ratio scores (*i.e.*, *HG* genes) by a fixed threshold of scores. As such, we select top 10% TFs for both yeast and human for further analysis, *i.e.*, 18 out of 177 yeast TFs and 16 out of 158 human TFs are selected as *HG* genes.

These 18 yeast TFs include YKL112W, YBR049C, YDR026C, YHR006W, YBL103C, YFR034C, YDR463W, YBL005W, YGL013C, YDR207C, YIR023W, YDL002C, YBL054W, YER088C, YNL216W, YJR127C, YLR375W and YML081W (in the descendent order of their Odds Ratio scores). Here, YKL112W, the gene with the highest score in yeast, directly mediates a number of different chromatin-related events such as DNA replication, gene silencing, chromatin remodeling and nucleotide excision repair. In addition, YKL112W transcriptionally regulates YIL072W (HOP1), which is a meiosis-specific protein required for chromosome synapsis [37]. YDR207C (top 10 ^*th*^ gene) is a key transcriptional regulator of early meiotic genes and it couples metabolic responses to nutritional cues with initiation and progression of meiosis. In particular, it interacts with YJR094C (IME1), which is a master regulator of meiosis, to activate transcription of early meiotic genes [38].

The top 16 human TFs are MYC, USF1, PRDM9, PLAG1, CUX1, TFAP4, TP53, TCF3, EP300, REST, RARA, INSM1, CTCF, PAX5, SP1 and ZIC2. We observe that the known *trans*−regulator PRDM9 (rank: 3 ^*rd*^) is captured by the Odds Ratio scores. Meanwhile, CTCF (rank: 13 ^*th*^) is a zinc finger protein that contains 11 *C*_2_*H*_2_−type zinc fingers (PRDM9 also belongs to a family of zinc finger proteins, with 14 *C*_2_*H*_2_−type zinc fingers). CTCF is annotated with terms, such as GO:0031060 (regulation of histone methylation), GO:0035065 (regulation of histone acetylation) and GO:0006306 (DNA methylation), plays a critical role in the epigenetic regulation [39].

We applied the gap score in Equation 6 for the GO term enrichment in yeast and human *HG* genes which are selected by Odds Ratio scores. Table 1 shows the top 10 GO terms enriched in yeast. The top GO terms of human are shown in Table S1 in our Additional file 1. As shown in the two tables, the top GO terms are related to epigenetic regulation, *i.e.*, chromatin remodeling, chromosome organization and histone modifications. Interestingly, these results of yeast and human are consistent with those of mouse in our previous studies [10],[36], showing that the epigenetic regulatory mechanism for recombination hotspots are conserved among multiple species.

**Table 1:** **GO terms enriched in yeast** ***HG* genes selected by Odds Ratio method**

**Rank**	**GO terms**	**GO term descriptions**	***gap***
1	GO:0007001	Chromosome organization and biogenesis (sensu Eukaryota)	0.312
2	GO:0006338	Chromatin remodeling	0.3
3	GO:0043044	ATP-dependent chromatin remodeling	0.287
4	GO:0042254	Ribosome biogenesis and assembly	0.284
5	GO:0042273	Ribosomal large subunit biogenesis and assembly	0.253
6	GO:0016575	Histone deacetylation	0.251
7	GO:0006333	Chromatin assembly or disassembly	0.242
8	GO:0006325	Establishment and/or maintenance of chromatin architecture	0.241
9	GO:0016577	Histone demethylation	0.239
10	GO:0006348	Chromatin silencing at telomere	0.208

#### 3.2.2 Genes prioritized by HB network alignment

We conduct GO term analysis for genes collected by the HB network alignment method (please refer to our previous study [11] for details on HB network construction and alignment). Due to the limited number of TF orthologs between yeast and human (or mouse), the HB network alignment method here is not applicable to the yeast TFs. We thus only show the results on human and mouse. Similarly we select top 10% TFs in each species for GO analysis, *i.e.*, 16 out of 158 human TFs and 15 out of 148 mouse TFs are selected. In particular, these *HG* genes in human are SP1, PRDM9, PAX5, ESR1, CTCF, NF1, NR6A1, MYOD1, YY1, USF1, PPARG, NFKB1, MYC, RELA, REL and MYOG in the decreasing order of their relevance scores. It is observed that 6 TFs are identified by both OR and HB methods, *i.e.*, MYC, PRDM9, SP1, CTCF, PAX5 and USF1.

Table 2 shows the top 10 GO terms enriched in the top 16 human *HG* genes (results for mouse *HG* genes are similar and thus are not shown here). Here, top 2 terms are quite interesting, namely GO:0007283 (spermatogenesis) and GO:0007276 (gamete generation). As we know, meiotic recombination hotspots play key roles in sexual reproduction. Our *HG* genes are enriched with functions highly related to “sexual reproduction”, and thus may perform their functions in the regulation of recombination hotspots. In addition, other top ranked terms are all epigenetic functions, which is consistent with the results collected by the Odds Ratio scores. Meanwhile, we also conducted GO analysis for 100 sets of random TFs. In contrast to the enrichment of epigenetic terms in Table 2, there are no epigenetic functions enriched in the random seeds as shown in Table S3 in our Additional file 1. It suggests that epigenetic functions are enriched in the *HG* genes selected by our two prioritizing methods but not enriched in the whole set of TFs.

**Table 2:** **GO terms enriched in human** ***HG* genes selected by HB network alignment method**

**Rank**	**GO terms**	**GO term descriptions**	***gap***
1	GO:0007283	Spermatogenesis	0.243
2	GO:0007276	Gamete generation	0.145
3	GO:0016568	Chromatin modification	0.122
4	GO:0051573	Negative regulation of histone H3-K9 methylation	0.119
5	GO:0006338	Chromatin remodeling	0.118
6	GO:0031060	Regulation of histone methylation	0.111
7	GO:0006337	Nucleosome disassembly	0.111
8	GO:0051574	Positive regulation of histone H3-K9 methylation	0.11
9	GO:0016584	Nucleosome positioning	0.108
10	GO:0051571	Positive regulation of histone H3-K4 methylation	0.108

#### 3.2.3 Genes from the KM method

We also selected 16 *HG* genes prioritized by newly designed KM method. As such, the genes prioritized by OR and KM have 10 in common, while those by HB and KM have 12 in common. The GO terms enriched in the genes prioritized by the KM method are shown in Table S2 in our Additional file 1. We observed that several epigenetic terms with high *gap* scores are enriched in those *HG* genes selected by our KM method. It is interesting that the terms GO:0007283, spermatogenesis (rank: 1^*st*^) and GO:0051573, negative regulation of histone H3-K9 methylation (rank: 11^*th*^) in Additional file 1: Table S2 have *gap* scores 0.312 and 0.136, respectively. In fact, the scores for these two terms in Additional file 1: Table S2 are higher than those in Table 2 (0.243 and 0.119 respectively), indicating that the two terms are more enriched in *HG* genes selected by KM method than HB method.

Next, we compute the semantic similarity between *HG* genes and two particular GO terms, *i.e.*, “DNA recombination” (GO:0006310) and “Meiosis” (GO:0007126), using Equation 5. These two terms are highly related to meiotic recombination hotspots. Table 3 shows the semantic similarity for *HG* genes, all the TFs with binding motifs and the whole set of human genes with GO annotations for comparison. It is observed that the *HG* genes (prioritized by OR, HB or KM) have higher average similarity to these two terms than other two sets of genes, indicating that our prioritizing methods are indeed helpful for selecting genes associated with recombination hotspots.

**Table 3:** Semantic similarity to two recombination related GO terms

**Species**	**Gene sets**	**DNA recombination**	**Meiosis**	**Average**
Mouse	HG genes (OR)	0.533	0.267	0.400
	HG genes (HB)	0.582	**0.288**	0.435
	HG genes (KM)	**0.603**	0.287	**0.445**
	148 TFs with binding motifs	0.526	0.211	0.368
	All mouse genes in GO	0.250	0.215	0.233
Human	HG genes (OR)	**0.577**	**0.291**	**0.434**
	HG genes (HB)	0.550	0.264	0.407
	HG genes (KM)	0.534	0.289	0.411
	158 TFs with binding motifs	0.506	0.220	0.363
	All human genes in GO	0.287	0.162	0.224
Yeast	HG genes (Odds Ratio)	**0.417**	0.181	**0.299**
	177 TFs with binding motifs	0.388	**0.201**	0.295
	All yeast genes in GO	0.309	0.190	0.250

The third and fourth column show the semantic similarity to ‘DNA recombination’ and ‘Meiosis’, respectively. The last column shows the average semantic similarity to these two terms. For each species, the values in bold are the highest similarity scores.

Obviously, the KM method achieves the higher average GO similarities than OR and HB methods for mouse as shown in Table 3. Meanwhile, the KM method for human is moderate—OR achieves the best performance and KM is slightly better than HB. In fact, several genes have high ranks by OR while they may have low ranks by HB. As a balance, KM generally will not select them as *HG* genes. EP300 and TCF3 in human are indeed such cases. They have high similarity to the two recombination related terms, *e.g.*, 0.548 for EP300 and 0.535 for TCF3, respectively. This would explain to some extent why KM does not achieve good results for human.

In addition, the *HG* genes in human collected by the OR method have higher similarity than those by the HB method, while the case for mouse is the opposite, *i.e.*, *HG* genes collected by the HB method have higher similarity than the OR method. This demonstrates that these two prioritizing methods may have their own advantages in different species. In the same species, they may also be complements to each other. For example in human, the gene YY1 has a low Odds Ratio score while it can be identified by HB network alignment method. It is a core component of the chromatin remodeling INO80 complex which is involved in transcriptional regulation, DNA replication and DNA repair. It is annotated with the terms GO:0006310 (DNA recombination) and GO:0000724 (double-strand break repair via homologous recombination) [40] and is involved in recombination events by binding to DNA recombination intermediate structures [41].

### 3.3 Pathway enrichment analysis for the prioritized genes

Besides the above GO analysis, we perform pathway enrichment analysis for our prioritized genes using various tools, including DAVID [42] (http://david.abcc.ncifcrf.gov), EnrichNet [43] (http://www.enrichnet.org) and WebGestalt [44] (http://bioinfo.vanderbilt.edu/webgestalt/analysis.php). We feed 16 human genes prioritized by the KM method to the above three tools and obtain the following results.

First, all the three tools demonstrated that the prioritized genes are enriched in cancer pathways, as well as the apoptosis pathway and MAPK signalling pathway. Out of 16 human prioritized genes, TP53, SP1 and MYC are well-known cancer genes that play crucial rules in genome instability. This would explain why our prioritized genes are enriched in cancer pathways. In addition, 5 and 3 out of 16 prioritized genes are in MAPK signalling pathway and apoptosis pathway respectively.

Second, EnrichNet reported an interesting KEGG pathway named “Homologous recombination”, which would be associated with our prioritized genes. Although the 14 genes involved in this pathway have no overlap with the prioritized genes, we found several links between our predicted trans-regulators and the homologous recombination pathway. For example, TP53 as one of the prioritized genes interacts with BRCA2 and RAD51, which are in the pathway of homologous recombination. They work together as key components for cell cycle control and DNA repair [45]. SP1 also interacts with BRCA2 and RAD51. Such links between the prioritized genes and the homologous recombination pathway would implicate that some of these genes are connected with recombination hotspots. The exact mechanism of such links needs further analysis, which however is beyond the scope of this paper.

### 3.4 Genes re-ranked by RWR

In the above subsection, the *HG* genes selected by various prioritizing methods (OR, HB and KM) are demonstrated to be enriched with epigenetic functions and have high similarity with two meiotic recombination-related GO terms. Here, we take them as “seeds” for the RWR algorithm in PPI networks and propagate their influence to other genes in the PPI networks, aiming to identify more genes related with meiotic recombination hotspots.

We focus on the analysis of those novel non-seed genes top-ranked by the RWR algorithm. Using the seeds selected by the OR method, Table 4 shows the top 10 non-seed genes in the PPI networks ranked by the RWR algorithm and their semantic similarity to terms GO:0006310 (DNA recombination) and GO:0007126 (Meiosis). Tables S4 and S5 in the Additional file 1 are similar while their seeds are selected by the HB and KM methods respectively. In these tables, we observed that top 10 non-seeds in human and yeast even have higher GO similarity than the seeds themselves. Meanwhile, top-ranked non-seeds in mouse have slightly lower or comparable GO similarity than seeds. As we know, seeds are selected based on direct evidence, *i.e.*, their binding to recombination hotspots. Meanwhile, the top-ranked non-seeds are collected from indirect evidence, *e.g.*, their physical interactions with seeds. Nevertheless, top-ranked non-seeds achieve high GO similarities with recombination related terms, implying the usefulness of PPI data for us to find and analyze individual genes for recombination hotspots.

**Table 4:** **Top genes ranked by the RWR algorithm (using** ***HG* genes with high Odds Ratio scores as seeds) and their average semantic similarity to two meiotic recombination related GO terms**

	**Yeast**		**Human**		**Mouse**	
**Rank**	**Genes**	**Similarity**	**Genes**	**Similarity**	**Genes**	**Similarity**
1	YDR176W	0.354	KPNA2	0.712	EP300	0.303
2	YNL118C	0.292	UBC	0.806	CREBBP	0.504
3	YBR155W	0.309	FBXW11	0.458	JUN	0.509
4	YDL076C	0.259	HDAC1	0.502	HCFC1	0.463
5	YKR086W	0.224	HDAC3	0.466	YWHAB	0.266
6	YGR200C	0.31	CREBBP	0.378	ESR1	0.482
7	YBR160W	0.659	GLI3	0.396	TAT	0.267
8	YLR103C	0.447	GLI1	0.307	RELA	0.396
9	YJR066W	0.608	GLI2	0.457	NFYB	0.37
10	YDR388W	0.144	SIN3A	0.67	SOX10	0.42
Average		0.361		0.515		0.398

**Figure 3:**
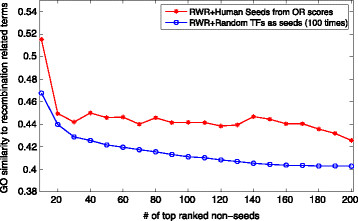
**The average GO similarities for genes top-ranked by RWR in BioGrid and BioGrid+PRDM9.** Figure 3 shows the average GO similarities for genes top-ranked by RWR in two PPI networks, *i.e.*, BioGrid and BioGrid+PRDM9 (BioGrid expanded with PRDM9).

We also test the performance of RWR using the random TFs as seeds. Figure 3 shows the GO similarities for top-ranked human non-seeds generated by random seeds and prioritized seeds respectively. Here, human seeds are prioritized by their OR scores and we generate random seeds (with the same size as prioritized seeds) for 100 times. In Figure 3, the average GO similarity of top-10 non-seeds generated by RWR with random seeds is even higher than that of our prioritized seeds. This indicates that RWR on PPI data may generate better candidate trans-regulatory genes than our prioritizing methods. More importantly, the combination of RWR and prioritizing methods achieves even better results, as top human non-seeds generated by prioritized seeds have higher GO similarities than those generated by random seeds as shown in Figure 3. Figure S1 in our Additional file 1 shows similar results for yeast.

Top-ranked non-seeds with high GO similarities as shown in above Table 4 and Figure 3 demonstrate that they are likely to be associated with recombination hotspots. We next show some cases which play important roles in recombination hotspots. Yeast gene YJR066W (rank: 9 ^*th*^) in Table 4 is a component of TORC1 complex and is involved in meiosis. Another yeast gene YBR160W (rank: 7 ^*th*^) is annotated with the following GO terms: GO:0006338 (chromatin remodeling), GO:0000706 (meiotic DNA double-strand break processing), GO:0051447 (regulation of meiotic cell cycle) and GO:0010569 (regulation of double-strand break repair via homologous recombination). Human KPNA2 in Table 4 as well as Additional file 1: Tables S4 and S5 is captured by RWR algorithm. It was previously reported to be involved in recombination, with a GO annotation GO:0000018 (regulation of DNA recombination) [46]. Human UIMC1 (rank: 1 ^*st*^) in Additional file 1: Table S4 is a component of BRCA1-A complex [47]. It has annotations including GO:0006302 (double-strand break repair), GO:0016568 (chromatin modification) and GO:0045739 (positive regulation of DNA repair). HDAC1 in Table 4, Additional file 1: Tables S4 and S5 is a component of the histone deacetylase complex and it is annotated with GO terms like GO:0006338 (chromatin remodeling) and GO:0006476 (protein deacetylation).

### 3.5 RWR in the PPI network expanded with PRDM9

As shown in the preceding section, many important genes associated with recombination hotspots can be identified by RWR in PPI networks. However, current protein interaction data for various species are still incomplete and noisy. For example, the well-known recombination regulator PRDM9 has no interaction records in BioGrid [34] or HPRD [48] databases. In order to propagate the influence of PRDM9 to other genes, its predicted interaction partners are collected from STRING [49] database, including SPO11, SPATA17, RNF212, H2AFX, H3F3A and H3F3B.

We obtained an expanded PPI network denoted as “BioGrid+PRDM9” (*i.e.*, by adding the interactions involving PRDM9 into current BioGrid). Using the seeds selected by the KM method, top 20 genes ranked by the RWR algorithm on the expanded PPI network are shown in Table 5. Similarly, Additional file 1: Tables S6 and S7 show top 20 genes using the seeds selected by OR and HB. In these 3 tables, we observed that these predicted interacting partners of PRDM9 generally have high ranks after running RWR algorithm. For example, SPO11, RNF212 [50] and H2AFX have high similarity to the two aforementioned recombination related terms, indicating that they are indeed involved in meiotic recombination. For SPATA17, since it has no annotation in GO, its similarity score is 0. However, it functions in meiosis as a spermatogenesis-associated protein.

**Table 5:** Top genes ranked by the RWR algorithm in an expanded PPI network “BioGrid+PRDM9” and their semantic similarity to two recombination related GO terms

	**BioGrid+PRDM9**		**BioGrid**	
**Rank**	**Genes**	**Similarity**	**Genes**	**Similarity**
1	KPNA2	0.712	KPNA2	0.712
2	UIMC1	0.464	UIMC1	0.464
3	FBXW11	0.458	FBXW11	0.458
4	UBC	0.806	UBC	0.806
5	EP300	0.548	EP300	0.548
6	SMARCA4	0.417	SMARCA4	0.417
7	SMAD3	0.438	SMAD3	0.438
8	HDAC1	0.502	HDAC1	0.502
9	**H2AFX**	0.838	POLR2A	0.425
10	POLR2A	0.425	SMAD2	0.432
11	**H3F3A**	0.301	CREBBP	0.378
12	SMAD2	0.432	RUNX1	0.392
13	CREBBP	0.378	SMAD4	0.457
14	RUNX1	0.392	SUMO1	0.432
15	SMAD4	0.457	DAXX	0.495
16	**H3F3B**	0.301	ID3	0.509
17	**SPO11**	0.883	MYB	0.568
18	**SPATA17**	0	PARP1	0.414
19	**RNF212**	0.668	RXRA	0.368
20	SUMO1	0.432	TAT	0.263
Average		0.493		0.474

The genes in bold are those predicted interaction partner of PRDM9 in STRING database.

**Figure 4:**
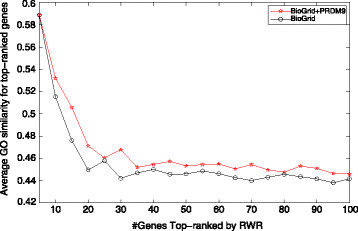
**GO similarities for top-ranked human non-seeds generated by random seeds and prioritized seeds respectively.** Figure 4 shows the average GO similarities for top-ranked human non-seeds generated by random seeds and prioritized seeds respectively.

Figure 4 shows the average GO similarities for those top-ranked genes in BioGrid as well as the expanded PPI network. Note that the seeds here for the RWR algorithm are selected by KM methods. Additional file 1: Figures S2 and S3 show the cases using seeds selected by OR and HB respectively. In Figure 4, a node (*x*, *y*) means that the top *x* genes have an average GO similarity *y*, *e.g.*, top 20 genes have average similarity of 0.493 and 0.474 in two PPI networks respectively as shown in Table 5. It is obvious that the top genes in the expanded network “BioGrid+PRDM9” have a higher average similarity than those in the original BioGrid. It is thus promising to identify recombination regulators by incorporating or predicting more protein-protein interactions for such well-known genes like PRDM9 in the future.

### 3.6 Protein complexes involved in recombination hotspots

With the genes identified as seeds by various methods (OR, HB and KM), we ran RWR and constructed PPI subnetworks, *e.g.*, with top 200 nodes. In this section, we will show the results using seeds selected by the HB method [11]. Here, we are still not able to detect protein complexes involving the PRDM9 protein after we added its 6 interaction partners into BioGrid (*i.e.*, SPO11, SPATA17, RNF212, H2AFX, H3F3A and H3F3B in the above section). This indicates that the expanded PPI network still does not have dense interaction structures around PRDM9. Therefore, it is desirable to find more interactions involving PRDM9 and its partners for further protein-complex detection.

Next, we will focus on the analysis of the complexes conserved between human and mouse. We exploit the Gene Ontology (GO) to evaluate the function enrichment of our conserved protein complexes based on p-values [22]. The p-value of a protein complex *C* with respect to a GO term *F* is defined in Equation 7.

(7) p−value=1−∑i=0k−1|F|i|V|−|F||C|−i|V||C|,

where *C* contains *k* proteins in *F* and |*V*| is the total number of proteins in a given genome. A predicted protein complex with low p-values indicates that it is enriched by proteins from the same functional group and thus statistically significant. In our experiments, all the conserved complexes are predicted to be significant, *i.e.*, with the lowest p-values lower than 0.01 [27]. This result shows that those conserved complexes are indeed enriched by common and specific functions.

**Figure 5:**
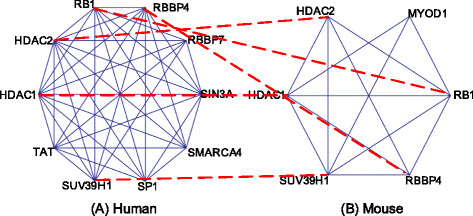
**Conserved complexes between human (A) and mouse (B) predicted by our pipeline.** Figure 5 shows a pair of complexes conserved between human **(A)** and mouse **(B)** predicted by our pipeline.

More specifically for the conserved complexes in human and mouse, we list top 5 GO terms with the lowest p-values for them. Figure 4 shows a conserved complex predicted by our pipeline. Meanwhile, Table 6 shows top 5 “cellular component” GO terms of the conserved complexes in Figure 5. Here, the Sin3 complex (GO:0016580) is a transcriptional repressor of protein-coding genes, through the gene-specific deacetylation of histones. The NuRD complex (GO:0016581) has ATP-dependent chromatin remodeling activity in addition to histone deacetylase (HDAC) activity. The ESC/E(Z) complex (GO:0035098) methylates lysine-27 and lysine-9 residues of histone H3.

**Table 6:** **Top-5 GO terms for the conserved complex predicted by NetPipe as shown in Figure**4

	**Human**		
**Rank**	**P-value**	**GO term**	**Term description**
1	6.53e-012	GO:0016580	Sin3 complex
2	5.65e-011	GO:0016581	NuRD complex
3	7.08e-009	GO:0035098	ESC/E(Z) complex
4	4.71e-008	GO:0000792	Heterochromatin
5	5.38e-007	GO:0005654	Nucleoplasm
	**Mouse**		
1	5.56e-010	GO:0016581	NuRD complex
2	3.89e-009	GO:0000792	Heterochromatin
3	1.86e-007	GO:0005654	Nucleoplasm
4	7.43e-007	GO:0016580	Sin3 complex
5	7.43e-007	GO:0035098	ESC/E(Z) complex

## 4 Conclusions

In this paper, we introduced a pipeline as shown in Figure 1 to identify genes and protein complexes associated with recombination hotspots. Based on the DNA-binding information of TFs, we introduced two complementary methods, *i.e.*, Odds Ratio scores (OR) and HB network alignment method (HB), to prioritize genes associated with recombination hotspots. We also proposed a ranking aggregation method called KM to combine the results from OR and HB. Furthermore, we exploited the PPI data to predict more proteins without binding information. Therefore, we effectively addressed the limitation that various prioritizing methods (OR, HB and KM) can only work for a small number of TFs with binding motifs. Meanwhile, we also detected protein complexes conserved between human and mouse that are associated with recombination hotspots. Evaluation results show that our pipeline is able to identify novel recombination-related genes and protein complexes. In addition, novel genes ranked in PPI networks have high semantic similarity to recombination related GO terms, showing PPI data are indeed a good source to select individual genes associated with recombination hotspots. For example, human protein KPNA2 is captured by RWR algorithm. It was previously reported to be involved in recombination, with a GO annotation GO:0000018 (regulation of DNA recombination) [46].

In our current results, we expanded the human PPI networks by including the interactions for PRDM9 and thus we managed to propagate the influence of PRDM9 to other proteins. However, we still cannot predict protein complexes involving PRDM9. The main reason for this issue could be that there are no dense interaction structures around PRDM9. Therefore, we may predict more interactions for PRDM9 and its partners by integrating multiple types of evidence. As such, there would be some dense structures around it, which can thus be detected by some existing density-based algorithms for protein complexes.

## Competing interests

The authors declare that they have no competing interests.

## Authors’ contributions

MW and JZ conceptualized and designed the method and drafted the manuscript together. MW was responsible for the implementation. CKK and XLL participated in discussion and conceptualization as well as revising the draft. All authors read and approved the manuscript.

## Additional file

## Supplementary Material

Additional file 1 Supplementary figures and tables. Click here for file
