# Supplementary material for: Finding trans-regulatory genes and protein complexes modulating meiotic recombination hotspots of human, mouse and yeast
Source: BMC Syst Biol. 2014 Sep 11;8:107. doi: 10.1186/s12918-014-0107-1 (PMC4236725; doi:10.1186/s12918-014-0107-1)
Supplement: Additional file 1 — Supplementary figures and tables. [file s12918-014-0107-1-S1.pdf]

Finding Trans-Regulatory Genes and Protein Complexes Modulating Meiotic Recombination Hotspots of Human, Mouse and Yeast

Min Wu, Chee-Keong Kwoh, Xiaoli Li and Jie Zheng

1 GO terms enrichment analysis

Table S1 shows top 10 GO terms that are enriched in the *HG* genes selected by the Odds Ratio scores (OR). It is obvious that epigenetic functions, especially histone modifications, are enriched in these genes.

Table S1. GO terms enriched in human *HG* genes selected by the Odds Ratio scores

Rank	GO terms	GO term descriptions	*gap*
1	GO:0051573	negative regulation of histone H3-K9 methylation	0.264
2	GO:0035067	negative regulation of histone acetylation	0.261
3	GO:0051572	negative regulation of histone H3-K4 methylation	0.259
4	GO:0051569	regulation of histone H3-K4 methylation	0.237
5	GO:0031060	regulation of histone methylation	0.236
6	GO:0045736	negative regulation of cyclin-dependent protein kinase activity	0.23
7	GO:0035065	regulation of histone acetylation	0.229
8	GO:0051574	positive regulation of histone H3-K9 methylation	0.226
9	GO:0051571	positive regulation of histone H3-K4 methylation	0.223
10	GO:0035066	positive regulation of histone acetylation	0.216

Table S2 shows top GO terms that are enriched in the *HG* genes selected by the KM method. We observed that some terms with high ranks are directly involving meiosis, e.g., GO:0007283 (spermatogenesis), GO:0007276 (gamete generation) and so on. In addition, some epigenetic terms are also enriched in these genes, for example, GO:0051573 (negative regulation of histone H3-K9 methylation) and GO:0051572 (negative regulation of histone H3-K4 methylation).

Epigenetic functions are enriched in the *HG* selected by various prioritizing methods (OR, HB and KM). Thus, we may doubt whether they are also enriched in the whole set of TFs. As such, we randomly generated 100 sets of TFs (each set with 16 TFs) and the top 10 GO terms with highest average *gap* scores are as shown in Table S3. It is obvious that there are no epigenetic functions in Table S3, indicating epigenetic functions enriched in *HG* genes while not enriched in the whole set of TFs.

Table S2. GO terms enriched in human *HG* genes with top odds ratio scores

Rank	GO terms	GO term descriptions	*gap*
1	GO:0007283	spermatogenesis	0.312
2	GO:0007276	gamete generation	0.199
3	GO:0045736	negative regulation of cyclin-dependent protein kinase activity	0.185
4	GO:0007128	meiotic prophase I	0.174
10	GO:0045814	negative regulation of gene expression, epigenetic	0.140
11	GO:0051573	negative regulation of histone H3-K9 methylation	0.136
15	GO:0051572	negative regulation of histone H3-K4 methylation	0.131
17	GO:0007143	female meiosis	0.130
19	GO:0019953	sexual reproduction	0.129
24	GO:0035067	negative regulation of histone acetylation	0.128

Table S3. GO terms enriched in random human TFs with top-10 average *gap* scores (over 100 random sets of seeds)

Rank	GO terms	GO term descriptions	Average *gap*
1	GO:0015695	organic cation transport	0.0326
2	GO:0048241	epinephrine transport	0.032
3	GO:0055085	transmembrane transport	0.0276
4	GO:0010248	establishment and/or maintenance of transmembrane electrochemical gradient	0.0276
5	GO:0000301	retrograde transport, vesicle recycling within Golgi	0.026
6	GO:0006891	intra-Golgi vesicle-mediated transport	0.0258
7	GO:0015909	long-chain fatty acid transport	0.0255
8	GO:0042953	lipoprotein transport	0.0254
9	GO:0015908	fatty acid transport	0.0254
10	GO:0046323	glucose import	0.0251

2 RWR with random seeds

In our main manuscript, we already show that top human non-seeds generated by prioritized seeds have higher GO similarity than those generated by random seeds. Here, Figure S1 shows the results for yeast. Similarly, yeast seeds are prioritized by their OR scores and we generate random seeds (with the same size as prioritized seeds) for 100 times. Figure S1 also demonstrates that top yeast non-seeds generated by prioritized seeds have higher GO similarity than those generated by random seeds. This result indicates once again that prioritized seeds are better than random seeds.

3 RWR results on BioGrid

Table S4 shows top 10 genes ranked by the RWR algorithm on the BioGrid data. The seeds for the RWR algorithm are selected by the HB method.

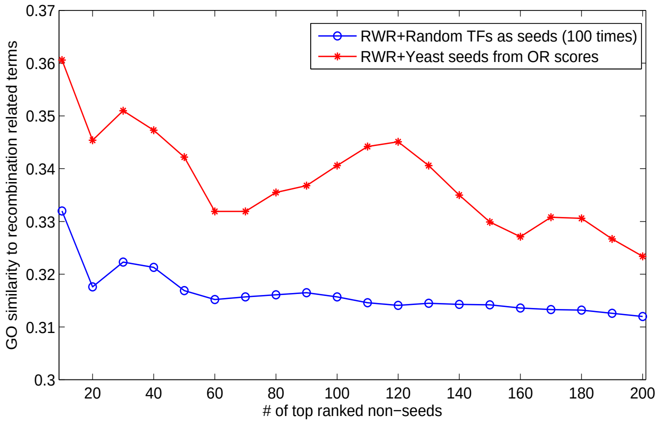

Fig. S1. GO similarity for top-ranked yeast non-seeds generated by random seeds and prioritized seeds respectively.

Table S4. Top genes ranked by the RWR algorithm (seeds are selected by the HB method) and their semantic similarity to two recombination related GO terms

	Human		Mouse	
Rank	Genes	Similarity	Genes	Similarity
1	UIMC1	0.464	CREBBP	0.504
2	UBC	0.806	EP300	0.303
3	EP300	0.548	SMAD3	0.438
4	SMARCA4	0.417	ID2	0.361
5	HDAC1	0.502	ID3	0.509
6	SMAD3	0.438	CALM1	0.378
7	CREBBP	0.378	RB1	0.439
8	POLR2A	0.425	SMAD4	0.457
9	SMAD2	0.432	TBP	0.37
10	KPNA2	0.712	TCF3	0.535
Average		0.512		0.429

Table S5 shows top 10 genes ranked by the RWR algorithm on the BioGrid data. The seeds for the RWR algorithm are selected by the KM method.

Table S5. Top genes ranked by the RWR algorithm (seeds are selected by the KM method) and their semantic similarity to two recombination related GO terms

	Human		Mouse	
Rank	Genes	Similarity	Genes	Similarity
1	KPNA2	0.712	EP300	0.303
2	UIMC1	0.464	CREBBP	0.504
3	FBXW11	0.458	JUN	0.509
4	UBC	0.806	RB1	0.439
5	EP300	0.548	RELA	0.396
6	SMARCA4	0.417	CEBPB	0.42
7	SMAD3	0.438	TBP	0.37
8	HDAC1	0.502	TRP53	0.575
9	POLR2A	0.425	NCOR2	0.397
10	SMAD2	0.432	GTF2I	0.37
Average		0.520		0.428

4 RWR results on BioGrid expanded with PRDM9

Figure S2 and Table S6 show the comparison results of RWR on two PPI networks, namely, BioGrid and BioGrid expanded with PRDM9 (BioGrid+PRDM9). Here, the seeds here are selected by the OR method.

Figure S3 and Table S7 show the comparison results of RWR on two PPI networks, namely, BioGrid and BioGrid expanded with PRDM9 (BioGrid+PRDM9). Please note that the seeds for the RWR algorithm are selected by the HB method.

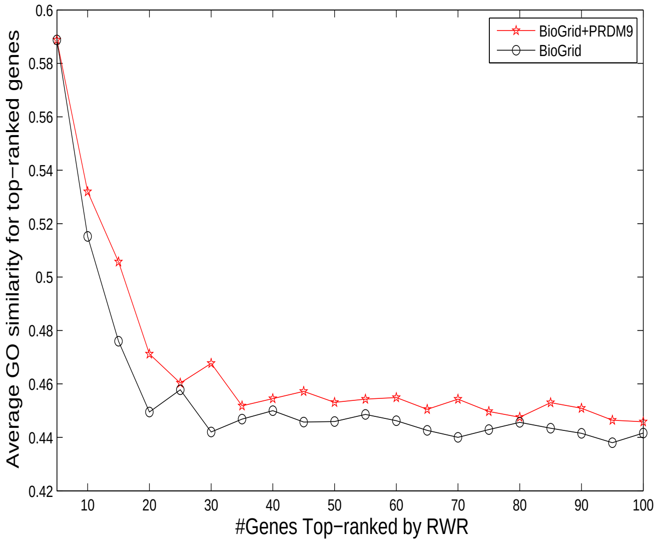

Fig. S2. The average GO similarity for genes top-ranked by RWR in BioGrid and BioGrid+PRDM9. The seeds for RWR are selected by the OR method.

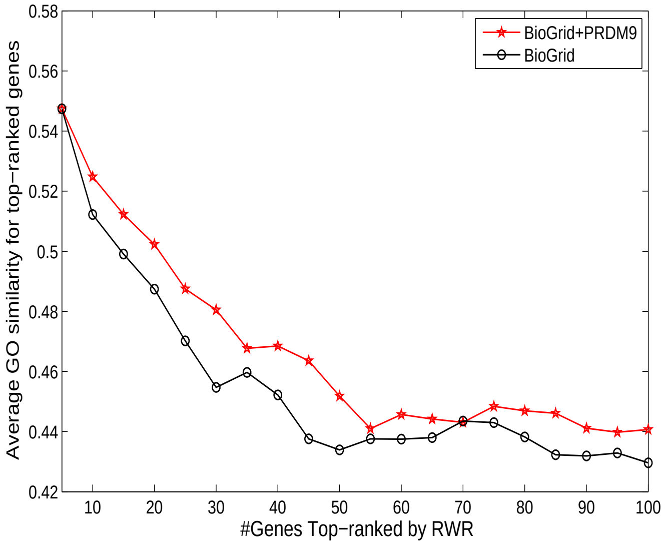

Fig. S3. The average GO similarity for genes top-ranked by RWR in BioGrid and BioGrid+PRDM9. The seeds for RWR are selected by the HB method.

Table S6. Top genes ranked by the RWR algorithm in an expanded PPI network "BioGrid+PRDM9" and their semantic similarity to two recombination related GO terms. The red genes are those predicted interaction partner of PRDM9 in STRING database. In addition, the seeds here are selected by the OR method.

	Human		Mouse	
Rank	Genes	Similarity	Genes	Similarity
1	KPNA2	0.712	KPNA2	0.712
2	UBC	0.806	UBC	0.806
3	FBXW11	0.458	FBXW11	0.458
4	HDAC1	0.502	HDAC1	0.502
5	HDAC3	0.466	HDAC3	0.466
6	CREBBP	0.378	CREBBP	0.378
7	GLI3	0.396	GLI3	0.396
8	GLI1	0.307	GLI1	0.307
9	GLI2	0.457	GLI2	0.457
10	H2AFX	0.838	SIN3A	0.67
11	SIN3A	0.67	HDAC2	0.446
12	HDAC2	0.446	CCND1	0.399
13	CCND1	0.399	KAT2B	0.42
14	KAT2B	0.42	TLE4	0.331
15	TLE4	0.331	RUNX1	0.392
16	RUNX1	0.392	TUBB3	0.569
17	TUBB3	0.569	SMARCA4	0.417
18	SMARCA4	0.417	RNF180	0.159
19	H3F3A	0.301	SORBS1	0.21
20	RNF180	0.159	DAXX	0.495
Average		0.471		0.450

Table S7. Top genes ranked by the RWR algorithm in an expanded PPI network "BioGrid+PRDM9" and their semantic similarity to two recombination related GO terms. The red genes are those predicted interaction partner of PRDM9 in STRING database. In addition, the seeds here are selected by the HB method.

	BioGrid+PRDM9		BioGrid	
Rank	Genes	Similarity	Genes	Similarity
1	UIMC1	0.464	UIMC1	0.464
2	UBC	0.806	UBC	0.806
3	EP300	0.548	EP300	0.548
4	SMARCA4	0.417	SMARCA4	0.417
5	HDAC1	0.502	HDAC1	0.502
6	SMAD3	0.438	SMAD3	0.438
7	CREBBP	0.378	CREBBP	0.378
8	H2AFX	0.838	POLR2A	0.425
9	POLR2A	0.425	SMAD2	0.432
10	SMAD2	0.432	KPNA2	0.712
11	KPNA2	0.712	TP53	0.575
12	H3F3A	0.301	SMAD4	0.457
13	TP53	0.575	RUNX1	0.392
14	SMAD4	0.457	ID3	0.509
15	RUNX1	0.392	SUMO1	0.432
16	ID3	0.509	DAXX	0.495
17	H3F3B	0.301	SRF	0.383
18	SPO11	0.883	MYB	0.568
19	SPATA17	0	RXRA	0.368
20	RNF212	0.668	HDAC2	0.446
Average		0.502		0.487
